# Empowering understanding: navigating consent to ketamine treatment in adolescent mental health

**DOI:** 10.3389/fpsyt.2024.1433348

**Published:** 2024-08-13

**Authors:** Aakash Sathappan, Benjamin Yudkoff

**Affiliations:** ^1^ Boston Children’s Hospital, Harvard Medical School, Boston, MA, United States; ^2^ Brigham and Women’s Hospital, Harvard Medical School, Boston, MA, United States; ^3^ Lumin Health, Boston, MA, United States

**Keywords:** ketamine, informed consent, adolescent, depression, ethics

## Abstract

The rising prevalence of mental health disorders in adolescents, compounded by treatment resistance, underscores the need for innovative interventions. Ketamine, recognized for its rapid antidepressant and anti-suicidal effects in adults, has recently emerged as a potential treatment for adolescents with treatment-resistant depression and suicidality. This paper aims to highlight key elements of the informed consent process, including obtaining parental consent and adolescent assent, and discussing the nature of ketamine treatment, its benefits, and potential risks. Obtaining informed consent for ketamine treatment in this demographic poses unique challenges. During the informed consent process, clinicians should balance an adolescent’s growing autonomy with parental consent and address the distinct features of treatment, including ketamine’s potential to induce psychedelic-like effects. Additionally, clinicians should highlight the “off-label” use in this population and the uncertainty inherent to treatment at this time, including the lack of data on repeated ketamine exposure on the developing brain. This paper also addresses challenging scenarios related to informed consent for this treatment, such as instances when parents are willing to consent but the adolescent refuses. Alternative treatment strategies such as transcranial magnetic stimulation (TMS) and electroconvulsive therapy (ECT) are also considered. In conclusion, while an emerging body of evidence suggests that ketamine shows potential for the acute treatment of adolescents with severe depression and suicidality, adherence to informed consent principles is paramount to ensure best clinical practices and uphold ethical standards amidst the current landscape of ongoing research.

## 1 Introduction

The prevalence of mental-health disorders among adolescents has increased significantly over the past decade. Between 2011 to 2021, the number of American high school students reporting persistent sadness or hopelessness with functional impairment increased from 28.5% to 42% (1). An estimated 16% of adolescents (ages 12-17) experienced at least one major depressive disorder - this cohort encompassed 2.7 million youth who reported suffering from severe major depression (2).

Despite the growing need for mental health support, over half of adolescents with major depression do not receive any form of treatment (2). Among those who have access to care approximately 1 in 5 are considered “treatment-resistant” after failing to find relief from their depression after undergoing the usual standard of care - two or more therapeutic trials of antidepressant medications (3). Adolescent depression is associated with functional morbidity including increased risk for substance use, exacerbation of physical illness, school avoidance, academic challenges, and elevated suicidality (4, 5). In 2021, an estimated 22% of all American high school students disclosed suicidal ideation with serious intent and nearly 1 in 4 high school females formulated a suicidal plan, with higher prevalence rates in LGBTQ+ youth (1).

Ketamine, a non-competitive antagonist of the N-methyl-D-aspartate (NMDA) receptor, has demonstrated rapid antidepressant and anti-suicidal effects in adults with treatment-resistant depression (6). Ketamine has also demonstrated potential therapeutic efficacy in the treatment of obsessive-compulsive disorder, anxiety disorders, post-traumatic stress disorder, and substance use disorders in adults (6). Ketamine appears to exert its antidepressant action through mechanisms involving glutamate neurotransmission, synaptic plasticity, neurogenesis via activation of downstream signaling pathways, and the up-regulation of mu-opioid receptors (7).

Ketamine has been historically used as an anesthetic and sedative agent in the pediatric populations (8). In recent years, a few studies suggest that subanesthetic doses of ketamine may be efficacious in the treatment of pediatric mood disorders, specifically treatment-resistant depression and suicidality in adolescents (9–11). These findings are preliminary and should be interpreted with caution as they merit replication with larger randomized controlled trials. Nevertheless, the increasing use of ketamine in clinical practice for off-label treatment of affective illness in adolescents reflects the growing psychiatric burden in this population and the need for more rapid-acting and efficacious treatments.

The medical informed consent process requires that clinicians provide patients information about their healthcare options. This involves disclosure around the nature of the diagnosis and proposed treatment, as well as the risks, benefits, and alternatives to treatment (12). Informed consent for an adolescent requires not only legal consent from parents but also voluntary assent from the minor, balancing respect for an adolescent’s growing autonomy and their role in the familial and legal structure (13). Ultimately, informed consent acts as a safeguard against harm, ensuring patients and families are treated with dignity and autonomy in medical settings.

Obtaining informed consent for use of ketamine in the adolescent population for the treatment of psychiatric disorders is an undefined area that poses unique challenges. Certain aspects of care, such as the potential for ketamine to induce distinct, psychedelic-like states are often difficult for the clinician to capture during the consent process. Other aspects of care, such as optimal dosing protocols and the long term safety profile of low-dose ketamine in adolescents are currently lacking a robust evidence base (11). This paper aims to highlight key elements of informed consent for ketamine use in adolescents with psychiatric illness, with the objective of advancing evidence-based clinical practices and maintaining ethical integrity within the context of limited empirical data.

## 2 Parental consent and adolescent assent

An adolescent’s assent, or their affirmative agreement to participate in care, is an important component of the informed consent process. A prerequisite to assent is an adolescent’s ability to demonstrate capacity to participate in treatment planning. This entails a clinician assess the adolescent’s understanding of the treatment and reflection on its significance in his/her life, their ability to communicate their decision-making rationale, and ability to consistently voice their preference to voluntarily proceed with or reject the treatment course (13)

The assent process requires a clinician to query the aforementioned information in age-appropriate manner and consider the adolescent’s social-emotional, cognitive, and language development. Although no clear threshold delineates when a minor becomes mature enough to assent to treatment, research suggests that normatively developing youth start demonstrating decision-making capacity similar to adults around the age of 14 (14). Hence, we proposed a minimum treatment age of 14 for youth interested in pursuing this treatment.

In cases involving cognitive impairments due to neurodevelopmental disorders or socio-emotional impairment due to trauma, for example, the assent process naturally becomes more complex and necessitates a more comprehensive capacity assessment. In these scenarios, it may be helpful to involve other professionals familiar with the adolescent, such as therapists, developmental specialists, and special education teachers to provide additional insights into their decision-making capacity.

Parental consent is typically mandatory for an adolescent to undergo any form of psychiatric treatment due to the adolescent’s inability to provide legal authorization independently (13). Given the novel and potent nature of these treatments, a supportive environment that extends beyond the medical treatment sessions is essential for adolescents to prepare for and integrate this potentially challenging experience. In some states that may allow minors to independently consent to ketamine treatment without parental approval, involving another trusted adult is advisable before proceeding with treatment.

In certain scenarios, conflicts may arise where parents are willing to consent to treatment but the adolescent refuses. In such cases, the concept of “set” adapted from classical psychedelic literature becomes relevant to navigating the informed consent process for ketamine - a molecule often categorized under the psychedelic umbrella. “Set” refers to an individual’s mindset, expectations, and intentions surrounding a psychedelic experience and is thought to significantly influence treatment outcomes (15). Respecting an adolescent’s decision to refuse ketamine treatment is imperative since proceeding with treatment against one’s wishes risks negatively impacting treatment experience and potentially causing a traumatizing and distressing experience from the outset. Moreover, it is considered ethically sound to honor an adolescent’s refusal of non-life-threatening care, although concerns about significant suicidality warrant careful consideration and dialogue with families and other clinicians (13). In situations involving imminent safety concerns, alternative treatment interventions such as inpatient hospitalization should be considered first. The decision to proceed with ketamine treatment against an adolescent’s wishes should be a last resort.

## 3 Nature of diagnosis & treatment

In order to effectively obtain both parental consent and adolescent assent a clinician must first provide the family with information regarding the nature of ketamine treatment which involves a comprehensive discussion on its benefits, risks, and alternatives. It is important to discuss with families that ketamine is not currently FDA-approved for the treatment of psychiatric disorders in minors, thereby categorizing its use in adolescent populations as “off-label”. Notably, the FDA has approved intranasal esketamine (Spravato) for use in treatment-resistant depression and depression with acute suicidality in adults in conjunction with an oral antidepressant (16). It should be emphasized that while off-label medication use is part of the standard of care for pediatric psychiatric disorders, special caution is warranted with ketamine due to its novel mechanism, modest yet growing evidence base, and potential to induce powerful psychedelic-like effects.

### 3.1 Nature of the treatment

Literature to date on the use of ketamine in adolescent populations is limited, consisting of a few case studies, an open-label study, and two randomized controlled trials focusing on adolescents with treatment-resistant depression and suicidality (9–11).

It may be helpful to share with families that the two recent randomized controlled trials have shown that ketamine confers rapid antidepressant and anti-suicidal effects in treatment-resistant depressed adolescents, although the long-term risk/benefit profile of ketamine remain largely unknown in this population (9, 10). Although statistics on treatment-emergent suicidality are limited, a recent randomized controlled trial in suicidal adolescents demonstrated significantly greater improvements in suicidal thoughts and depressive symptoms in the IV esketamine group compared to the IV midazolam placebo group after one week (10). Clinicians must confidently convey this uncertainty to families, delineating which aspects of care are informed by existing literature and which are based on their clinical judgment and experience.

Ideal induction and maintenance protocols for dosing are yet to be established, but typically studies in adolescents have employed IV racemic ketamine at a dose of 0.5 mg/kg over 40 minutes, consistent with standard dosing protocols in adult studies (11).

While other disorders in youth such as obsessive-compulsive disorder, anxiety disorders, and PTSD may respond to ketamine treatment, we recommend using ketamine exclusively for treatment-resistant depression or suicidality, as these indications are best supported by the current literature.

### 3.2 Risks/benefits/alternatives

Ketamine and esketamine are associated with transient increases in blood pressure and heart rate in patients (10-50%), peaking at 40 minutes and returning to baseline within two to four hours (17, 18). Adolescents should anticipate monitoring of blood pressure throughout the experience and post-session to ensure vital sign normalization. Other common side effects such as dissociation (36-72%), vertigo (25%), sedation (22.1%), dizziness (20.4%), nausea (16.4%) should also be reviewed (18, 19). Exclusionary criteria for medical and psychiatric conditions should be informed by the adult literature, including factors such as uncontrolled hypertension, aneurysmal disease, arteriovenous malformations, recent head trauma, stroke, porphyria, liver disease, kidney disease, untreated thyroid disease, and pregnancy for medical comorbidities and primary psychotic disorders for psychiatric comorbidities (20).

An adolescent should be able to convey their understanding that he or she may encounter a range of emotions during treatment, from awe and euphoria to fear and panic. Indeed, a clinician should attempt to capture the “ineffable” nature of the ketamine experience and encourage an adolescent to approach the treatment in a curious and non-judgmental manner. Of note, while dissociative and perceptual changes, including distortions in time and space, are common during ketamine experiences, they are generally tolerable and tend to diminish within two hours.

Nevertheless, it is crucial to review protocols with patients and their families for how to manage panic, dysphoria, or suicidal thoughts that may occur within the treatment setting. Providers should discuss and rehearse with an adolescent the utilization and preference of supportive care strategies in crisis scenarios. These may include interventions such as reassurance, grounding techniques, and offering options to re-establish a calm environment such as a weighted blanket or relaxing music. Pharmacological interventions should also be discussed and consented including administration of a benzodiazepine for acute anxiety and antipsychotics for severe and distressing dissociation or psychotic symptoms. Parents should also be informed that adolescents are required to remain in the treatment setting for the entirety of the experience to ensure their physical and psychological safety. A standardized suicide risk assessment should be conducted if suicidal ideation emerges. For suicidal ideation that persists or worsens a crisis intervention protocol should be discussed and implemented such as an extended observation period with a care companion or admittance of the adolescent to a higher level of care if warranted.

Ketamine treatment also holds the potential to modify cognitive biases and schemas in adolescents. Unlike antidepressants and psychotherapy, which produce similar effects at a slower pace, ketamine can prompt these changes rapidly though these changes are believed to be less robust (21). Nevertheless, these acute alterations to an adolescent’s temperament and personality may be perceived variably depending on the individual and environmental milieu. For instance, ketamine treatment may empower adolescents, enabling them to overcome entrenched beliefs and adopt new perspectives. Conversely, inadequate peer and social support may lead to destabilization in the face of these changes.

Discussion with parents should include the possibility of treatment-emergent, recreational ketamine abuse, although such cases have not been reported in the literature to date (18, 20). It is crucial to emphasize that active substance abuse precludes adolescents from treatment. Intravenous (IV) or intramuscular (IM) dosing is recommended over oral or sublingual dosing to ensure medical supervision and minimize the risk of diversion or abuse. Given some of the particular risk considerations unique to this vulnerable class of patients, it is recommended that at home use of ketamine be limited to extraordinary circumstances.

It’s also important to convey to families that the long-term effects of ketamine on the developing adolescent brain are uncertain. Animal model data suggest chronic exposure to standard clinical doses (0.5 -1 mg/kg) may disrupt brain maturation and cognitive function (22). Moreover, ketamine toxicity sequelae, such as liver injury, cystitis, and cognitive impairment, are documented in long-term abuse cases but not evident with standard clinical dosing (23). At this time, we recommend offering a short treatment course (~1-2 months) given the uncertainty of repeated ketamine exposure on adolescent neurodevelopment. Additionally, a clinician must discuss alternative treatments such as electroconvulsive therapy (ECT) and transcranial magnetic stimulation (TMS). These interventions presently boast a stronger evidence base compared to ketamine and are FDA-approved for adolescents with treatment-resistant depression (24). However, discussion should also include the risks of ECT and TMS, such as the cognitive deficits of ECT and the more involved treatment course of daily TMS treatment compared to weekly or twice-weekly ketamine treatment (24, 25). Other treatment modalities such as psychopharmacological augmentation strategies, participation in partial hospital programs and/or residential programs should also be part of the consent process.

## 4 Discussion

Ketamine and its derivative esketamine have recently emerged as potential interventions for adolescents experiencing severe depression and suicidality. It is important to highlight, however, that the current studies have several limitations. Existing studies in adolescents have only examined short-term effects, with follow-up periods typically lasting 2-4 weeks, leaving long-term efficacy and safety largely unknown (9, 10). The power of these studies is limited by small sample sizes, with the largest randomized controlled trial including 54 participants (9). The absence of standardized outcome measures across studies as well as heterogeneity in dosing parameters, treatment setting, and route of administration complicates direct comparisons between studies and limits generalizability. Moreover, most studies have focused on treatment resistant depression, with limited data on other conditions or comorbidities common in adolescents. Finally, although both randomized controlled trials employed midazolam as an active placebo control, the acute dissociative effects of ketamine were difficult to mask, compromising the integrity of these blinded study designs (9, 10). These limitations highlight the need for larger trials with longer follow-up periods and standardized protocols to better understand the efficacy, safety, and optimal use of ketamine in adolescent mental health treatment.

Despite the limited body of evidence, ketamine treatment is increasingly finding its place in clinical practice. Ethical considerations naturally arise: should this treatment be withheld until it garners regulatory approval and more robust support from randomized controlled clinical trials? Alternatively, is it justifiable to offer these treatments to adolescents who have shown inadequate responses to previous medication trials without significant improvement, and are demonstrating substantial functional decline, such as school avoidance and suicidality?

During this time of uncertainty, it behooves clinicians who decide to offer this treatment to navigate the informed consent process thoughtfully (**Table 1**). The assent process, tailored to the adolescent’s age, emotional state, and cognitive abilities, should emphasize the uncertainty inherent to the ketamine treatment course. Offering a second session for discussion solely with the adolescent reinforces their emerging autonomy and helps ensure their assent is voluntary and free of coercion. It may also be helpful to leverage the adolescent’s natural inclination to connect with peers and create support groups as a way to help one prepare for and integrate an experience that is difficult to capture in words. High-quality informational videos can enhance engagement and facilitate standardized discussions around informed consent. Utilizing quizzes and teach-back methods can further ensure comprehension and engagement.

**Table 1:** Navigating the informed consent process.

Challenges	Possible Solutions
Assess Adolescent’s Capacity for Decision Making	• Use age-appropriate language, avoid medical jargon • Consider adolescent’s cognitive, language, and socio-emotional development in addition to chronological age • May be helpful to involve other professionals familiar with adolescent to provide additional insights into decision-making capacity • Offer separate discussion with adolescent to promote emerging autonomy • Evaluate adolescent’s ability to maintain consistent choice over time through sequential check-ins during treatment course
Adolescent Dissent	• Respect an adolescent’s refusal of treatment to avoid negative treatment experience • For adolescent refusal of treatment in face of imminent, life-threatening suicidality obtain consultation with other clinicians. • Consider other alternatives such as ECT, TMS, and higher levels of care
Discussing Nature of Ketamine Experience	• Provide examples of possible effects including sudden perceptual changes and distortions of time and space • Emphasize that while most of these changes are time-limited to the treatment duration, one may experience changes in mood and beliefs that may persist after the treatment session • Encourage participants to approach the treatment session with a curious, non-judgemental approach
Addressing Potential Side Effects/Risk	• Review common side effects (blood pressure changes, nausea, sedation, discoordination, altered sensorium) • Discuss and rehearse protocols for managing adverse reactions • Recommend short treatment course (1-2 months) due to limited data on long-term safety and efficacy

An unprecedented aspect of the informed consent process for ketamine treatment in adolescents is its potential to induce rapid, profound changes in mood and belief systems akin to psychedelics. This presents a dilemma: Are we impeding adolescents’ development of resilience by providing such a potent treatment? Alternatively, could ketamine serve as a catalyst for resilience, offering hope for a brighter future?

In cases of treatment-resistant depression or suicidality with severe functional impairment, the risks associated with untreated illness may surpass those posed by ketamine treatment. Furthermore, clinicians can mitigate the potential risks of ketamine treatment by offering a short treatment course with frequent re-evaluation to assess efficacy and safety. It is possible that through thoughtful messaging and reflection during the informed consent process that ketamine treatment may support recovery and restore functioning for adolescents grappling with treatment-resistant depression and suicidality.

## Data Availability

The original contributions presented in the study are included in the article/supplementary material. Further inquiries can be directed to the corresponding author/s.

## References

[1] Youth Risk Behavior Survey Data Summary & Trends Report: 2011-2021. Available online at: https://www.cdc.gov/healthyyouth/data/yrbs/yrbs_data_summary_and_trends.htm (Accessed May 15, 2024).

[2] Reinert M Nguyen T Fritze D. The State of Mental Health in America 2023. (2022). Alexandria, VA: Mental Health America. Available online at: https://mhanational.org/sites/default/files/2023-State-of-Mental-Health-in-America-Report.pdf (Accessed May 15, 2024).

[3] Vitiello B Emslie G Clarke G Wagner KD Asarnow JR Keller M. Long-term outcome of adolescent depression initially resistant to SSRI treatment. J Clin Psychiatry. (2011) 72:388–96. doi: 10.4088/JCP.09m05885blu PMC3070064 21208583

[4] Fergusson DM Woodward LJ. Mental health, educational, and social role outcomes of adolescents with depression. Arch Gen Psychiatry. (2002) 59:225–31. doi: 10.1001/archpsyc.59.3.225 11879160

[5] Weissman MM Wolk S Goldstein RB Moreau D Adams P Greenwald S. Depressed adolescents grown up. JAMA. (1999) 281:1707–13. doi: 10.1001/jama.281.18.1707 10328070

[6] Walsh Z Mollaahmetoglu OM Rootman J Golsof S Keeler J Marsh B. Ketamine for the treatment of mental health and substance use disorders: comprehensive systematic review. BJPsych Open. (2022) 8:e19. doi: 10.1192/bjo.2021.1061 PMC8811778 35040425

[7] Hess EM Riggs LM Michaelides M Gould TD. Mechanisms of ketamine and its metabolites as antidepressants. Biochem Pharmacol. (2022) 197:114892. doi: 10.1016/j.bcp.2021.114892 34968492 PMC8883502

[8] Dolansky G Shah A Mosdossy G Rieder M. What is the evidence for the safety and efficacy of using ketamine in children? Paediatr Child Health. (2008) 13:307–8. doi: 10.1016/S2215-0366(17)30272-9 PMC2529431 19337601

[9] Dwyer JB Landeros-Weisenberger A Johnson JA Londono Tobon A Flores JM Nasir M. Efficacy of intravenous ketamine in adolescent treatment-resistant depression: A randomized midazolam-controlled trial. Am J Psychiatry. (2021) 178:352–62. doi: 10.1176/appi.ajp.2020.20010018 33653121

[10] Zhou Y Lan X Wang C Zhang F Liu H Fu L. Effect of repeated intravenous esketamine on adolescents with major depressive disorder and suicidal ideation: A randomized active-placebo-controlled trial. J Am Acad Child Adolesc Psychiatry. (2024) 63:507–18. doi: 10.1016/j.jaac.2023.05.031 37414272

[11] Ryan K Hosanagar A. Ketamine use in child and adolescent psychiatry: emerging data in treatment-resistant depression, insights from adults, and future directions. Curr Psychiatry Rep. (2023) 25:337–44. doi: 10.1007/s11920-023-01432-w 37389787

[12] Appelbaum PS. Informed consent to psychedelic treatment—A work in progress. JAMA Psychiatry. (2024) 81(6):543–4. doi: 10.1001/jamapsychiatry.2024.0124 38598204

[13] Katz AL Webb SA COMMITTEE ON BIOETHICS Macauley RC Mercurio MR Moon MR. Informed consent in decision-making in pediatric practice. Pediatrics. (2016) 138:e20161485. doi: 10.1542/peds.2016-1485 27456510

[14] Field MJ Behrman RE Children I of M (US) C on CRI. Understanding and agreeing to children’s participation in clinical research, in: Ethical Conduct of Clinical Research Involving Children (2004). National Academies Press (US. Available online at: https://www.ncbi.nlm.nih.gov/books/NBK25560/ (Accessed May 10, 2024). 20669469

[15] Marks M Brendel RW Shachar C Cohen IG. Essentials of informed consent to psychedelic medicine. JAMA Psychiatry. (2024). doi: 10.1001/jamapsychiatry.2024.0184 38598209

[16] Bahr R Lopez A Rey JA. Intranasal esketamine (SpravatoTM) for use in treatment-resistant depression in conjunction with an oral antidepressant. Pharm Ther. (2019) 44:340–75. PMC6534172 31160868

[17] Liebe T Li S Lord A Colic L Krause AL Batra A. Factors influencing the cardiovascular response to subanesthetic ketamine: A randomized, placebo-controlled trial. Int J Neuropsychopharmacol. (2017) 20:909–18. doi: 10.1093/ijnp/pyx055 PMC5737852 29099972

[18] Daly EJ Singh JB Fedgchin M Cooper K Lim P Shelton RC. Efficacy and safety of intranasal esketamine adjunctive to oral antidepressant therapy in treatment-resistant depression: A randomized clinical trial. JAMA Psychiatry. (2018) 75:139–48. doi: 10.1001/jamapsychiatry.2017.3739 PMC5838571 29282469

[19] Short B Fong J Galvez V Shelker W Loo CK. Side-effects associated with ketamine use in depression: a systematic review. Lancet Psychiatry. (2018) 5:65–78. doi: 10.1016/S2215-0366(17)30272-9 28757132

[20] McIntyre RS Rosenblat JD Nemeroff CB Sanacora G Murrough JW Berk M. Synthesizing the evidence for ketamine and esketamine in treatment-resistant depression: an international expert opinion on the available evidence and implementation. Am J Psychiatry. (2021) 178:383–99. doi: 10.1176/appi.ajp.2020.20081251 PMC9635017 33726522

[21] Bottemanne H Morlaas O Claret A Sharot T Fossati P Schmidt L. Evaluation of early ketamine effects on belief-updating biases in patients with treatment-resistant depression. JAMA Psychiatry. (2022) 79:1124–32. doi: 10.1001/jamapsychiatry.2022.2996 PMC9520441 36169969

[22] Zimmermann KS Richardson R Baker KD. Esketamine as a treatment for paediatric depression: questions of safety and efficacy. Lancet Psychiatry. (2020) 7:827–9. doi: 10.1016/S2215-0366(19)30521-8 31952957

[23] Strous JFM Weeland CJ van der Draai FA Daams JG Denys D Lok A. Brain changes associated with long-term ketamine abuse, A systematic review. Front Neuroanat. (2022) 16:795231. doi: 10.3389/fnana.2022.795231 35370568 PMC8972190

[24] Ledesma-Corvi S Jornet-Plaza J Gálvez-Melero L García-Fuster MJ. Novel rapid treatment options for adolescent depression. Pharmacol Res. (2024) 201:107085. doi: 10.1016/j.phrs.2024.107085 38309382

[25] Croarkin PE Elmaadawi AZ Aaronson ST Schrodt GR Holbert RC Verdoliva S. Left prefrontal transcranial magnetic stimulation for treatment-resistant depression in adolescents: a double-blind, randomized, sham-controlled trial. Neuropsychopharmacology. (2021) 46:462–9. doi: 10.1038/s41386-020-00829-y PMC7852515 32919400

